# Non-Invasive Ventilation Strategies in Children With Acute Lower Respiratory Infection: A Systematic Review and Bayesian Network Meta-Analysis

**DOI:** 10.3389/fped.2021.749975

**Published:** 2021-12-02

**Authors:** Zhili Wang, Yu He, Xiaolong Zhang, Zhengxiu Luo

**Affiliations:** ^1^Department of Respiratory Medicine, Children's Hospital of Chongqing Medical University, National Clinical Research Center for Child Health and Disorders, Ministry of Education Key Laboratory of Child Development and Disorders, Chongqing Key Laboratory of Pediatrics, Chongqing, China; ^2^Department of Pediatrics, Jiangjin District Central Hospital, Chongqing, China

**Keywords:** acute lower respiratory infection, BIPAP, children, CPAP, HFNC, meta-analysis, non-invasive ventilation

## Abstract

**Background:** Multiple non-invasive ventilation (NIV) modalities have been identified that may improve the prognosis of pediatric patients with acute lower respiratory infection (ALRI). However, the effect of NIV in children with ALRI remains inconclusive. Hence, this study aimed to evaluate the efficacy of various NIV strategies including continuous positive airway pressure (CPAP), high flow nasal cannula (HFNC), bilevel positive airway pressure (BIPAP), and standard oxygen therapy in children with ALRI and the need for supplemental oxygen.

**Methods:** Embase, PubMed, Cochrane Library, and Web of Science databases were searched from inception to July 2021. Randomized controlled trials (RCTs) that compared different NIV modalities for children with ALRI and the need for supplemental oxygen were included. Data were independently extracted by two reviewers. Primary outcomes were intubation and treatment failure rates. Secondary outcome was in-hospital mortality. Pairwise and Bayesian network meta-analyses within the random-effects model were used to synthesize data. The certainty of evidence was assessed using the Grading of Recommendations Assessment, Development and Evaluation framework.

**Results:** A total of 21 RCTs involving 5,342 children were included. Compared with standard oxygen therapy, CPAP (OR: 0.40, 95% CrI: 0.16–0.90, moderate quality) was associated with a lower risk of intubation. Furthermore, both CPAP (OR: 0.42, 95% CrI: 0.19–0.81, low quality) and HFNC (OR: 0.51, 95% CrI: 0.29–0.81, low quality) reduced treatment failure compared with standard oxygen therapy. There were no significant differences among all interventions for in-hospital mortality. Network meta-regression showed that there were no statistically significant subgroup effects.

**Conclusion:** Among children with ALRI and the need for supplemental oxygen, CPAP reduced the risk of intubation when compared to standard oxygen therapy. Both CPAP and HFNC were associated with a lower risk of treatment failure than standard oxygen therapy. However, evidence is still lacking to show benefits concerning mortality between different interventions. Further large-scale, multicenter studies are needed to confirm our results.

**Systematic Review Registration**: https://www.crd.york.ac.uk/prospero/display_record.php?RecordID=172156, identifier: CRD42020172156.

## Introduction

Acute lower respiratory infection (ALRI), such as pneumonia and bronchiolitis, constitute a major cause of respiratory failure in children (1, 2), of whom 15% may need intensive care unit (ICU) care and respiratory support (3). The global mortality annually is estimated as 6,50,000 for ALRI in children younger than 5 years (4). Standard oxygen therapy (SOT) at a flow rate of 1–4 L/min for children with ALRI and hypoxemia is recommended by the World Health Organization (WHO) (5, 6). However, despite the provision of SOT and supportive care, more effective respiratory support systems are still needed for some critically ill children because of worsening respiratory distress (7).

To avoid airway invasivity related to the intubation, various non-invasive ventilation (NIV) strategies, such as continuous positive airway pressure (CPAP), bilevel positive airway pressure (BIPAP) ventilation, and high flow nasal cannula (HFNC), have been proposed among pediatric patients (8, 9). However, the effect of NIV in children with ALRI remains inconclusive. A previous pairwise meta-analysis (10) that compared two of the three respiratory support devices (SOT, CPAP, and HFNC) revealed that in terms of reducing treatment failure, HFNC was better than SOT but inferior to CPAP. However, there were no significant differences between the therapies in terms of intubation and mortality rates. However, this conventional meta-analysis only focused on head-to-head trials of comparison of two interventions without assessing multiple interventions as a whole. In addition, since the publication of this meta-analysis, several new randomized controlled trials (RCTs) have been published (11–18). More importantly, there are no systematic reviews that have assessed the efficacy of BIPAP in pediatric patients.

A network meta-analysis (NMA) enables comparison of multiple interventions and improved precision through a combination of direct and indirect estimates of effects (19, 20). Therefore, we aimed to perform a rigorous and comprehensive Bayesian network meta-analysis to update current clinical study data and evaluate the effect of multiple NIV strategies in children with ALRI.

## Methods

This systematic review was registered on PROSPERO with registration number: CRD42020172156. This report complies with the recommendations of Preferred Reporting Items for Systematic Review and Meta-Analysis (PRISMA) Extension statement for NMA (21).

### Search Strategy

Pubmed, Embase, Cochrane Central Register of Controlled Trials (CENTRAL), and Web of Science were searched by one reviewer (ZW) from inception to August 1, 2020 (full search strategy is listed in Supplementary Material). The literature search was last updated on July 1, 2021.

### Eligibility Criteria and Outcome Measures

#### Type of Studies

We included all RCTs reported in English. Observational studies, case reports, review articles, comments, letters, conference abstracts, and editorials were excluded.

#### Type of Participants

This review included children aged from 29 days to 18 years, with ALRI (including bronchiolitis and WHO-defined pneumonia or severe pneumonia) and the need for supplemental oxygen. Following the WHO guideline, pneumonia is defined as acute presentation of either cough or tachypnea and also had either tachypnea or lower chest wall indrawing (6). WHO-defined severe pneumonia is based on cough or difficulty in breathing plus at least one of the following: central cyanosis or oxygen saturation <90% on pulse oximetry; respiratory distress (e.g., grunting, chest indrawing); and general danger signs (inability to breastfeed or drink, lethargy or unconscious, and convulsions) (6). The definitions of bronchiolitis were individualized for each study.

Studies conducted in pediatric intensive care unit (PICU), general ward, or emergency department (ED) were included. Studies that only include patients in the neonatal period (the first month of life) were excluded. We excluded treatments used at home or for chronic conditions.

#### Types of Interventions and Comparators

We included RCTs comparing two or more of the following four non-invasive respiratory support devices: (1) SOT: nasal cannula, nasal prong, nasal catheter, and mask with no limit on the flow rate; (2) CPAP: the ventilatory setting and interface were not limited; (3) HFNC: the flow rate and fraction of inspired oxygen (FiO_2_) were not limited; and (4) BIPAP: the ventilatory setting and interface were not limited.

#### Type of Outcomes

The primary outcomes were (1) treatment failure, defined by the individual authors in the included studies, and (2) the rate of intubation. The secondary outcome was in-hospital mortality at the end of the follow-up period (<28 days).

#### Study Selection and Data Extraction

Two reviewers (YH and XZ) independently screened the titles and abstracts retrieved from the search strategy to identify those meeting the pre-specified criteria. Subsequently, further screening was performed to select eligible articles by reviewing the full texts.

The two reviewers (YH and XZ) performed data extraction independently. The following data were extracted: first author, publication year, country, study characteristics (trial design, sample size, study setting, and funding source), patients' characteristics [age, diagnosis information, comorbidities, and mean pulse oximetry saturation (SpO_2_) on admission], details of the intervention, and outcome data for each endpoint of interest. Any disagreement was resolved by discussion.

#### Risk of Bias Assessment

Two researchers (YH and XZ) evaluated the methodological quality of the eligible studies according to the Cochrane collaboration's risk of bias tool for randomized trials (22). Disagreements were solved by consensus.

### Statistical Analysis

#### Pairwise Meta-Analysis

Conventional pairwise meta-analysis was conducted using the Mantel-Haenszel method within a random-effects model. Odds ratios (ORs) and 95% confidence intervals (CIs) were calculated for all dichotomous outcomes using Review Manager software (RevMan version 5.3). Heterogeneity was assessed using the *I*^2^ statistic (low heterogeneity = 25%, moderate heterogeneity = 50%, and high heterogeneity = 75%) (23).

#### Network Meta-Analysis

The NMA was conducted in a Bayesian framework random-effects model (24) using the Markov Chain Monte Carlo with vague priors by “GeMTC” package in R (version 3.6.2) and WinBUGS (version 1.4.3); the results were presented in the form of ORs and 95% credibility intervals (CrIs). The analyses used generalized linear models with a logit link function and 100,000 iterated simulations discarding the initial 20,000 iterations as burn-in. *I*^2^ statistic was used to detect global heterogeneity by using the “mtc.anohe” function of the “GeMTC” package (25). The node-splitting method was used to assess inconsistency between direct and indirect comparisons (26). Surface under the cumulative ranking curve (SUCRA) was calculated to rank the probabilities for each intervention, and a larger value indicates the better rank for each treatment (27). When 10 or more studies were available for an outcome, we assessed publication bias using the comparison-adjusted funnel plots in Stata (version 15.1).

The certainty of the evidence for the each outcome was evaluated using the Grading of Recommendations Assessment, Development and Evaluation (GRADE) framework (28) with five criteria: study limitations, imprecision, indirectness, heterogeneity and inconsistency, and publication bias.

#### Network Meta-Regression, Subgroup Analysis, and Sensitivity Analysis

Network meta-regression and subgroup analyses [considering mean age (<1 year vs. more than 1 year), country income (high income vs. low and middle income), mean SpO_2_ on admission (lower than 92% vs. more than 92%), study location (ICU vs. non-ICU), and type of disease (pneumonia vs. bronchiolitis)] were performed to explain the observed between-trail heterogeneity if data were available. For the sensitivity analyses, we eliminated the study by McCollum et al. (18). from consideration as the study included participants with high-risk conditions (such as HIV infection and severe malnutrition), and it was conducted in the general ward without daily physician supervision. Therefore, the lack of physician oversight and the examined populations were significantly different from the other studies.

## Results

### Selection of Articles and Characteristics of Studies

**Figure 1:**
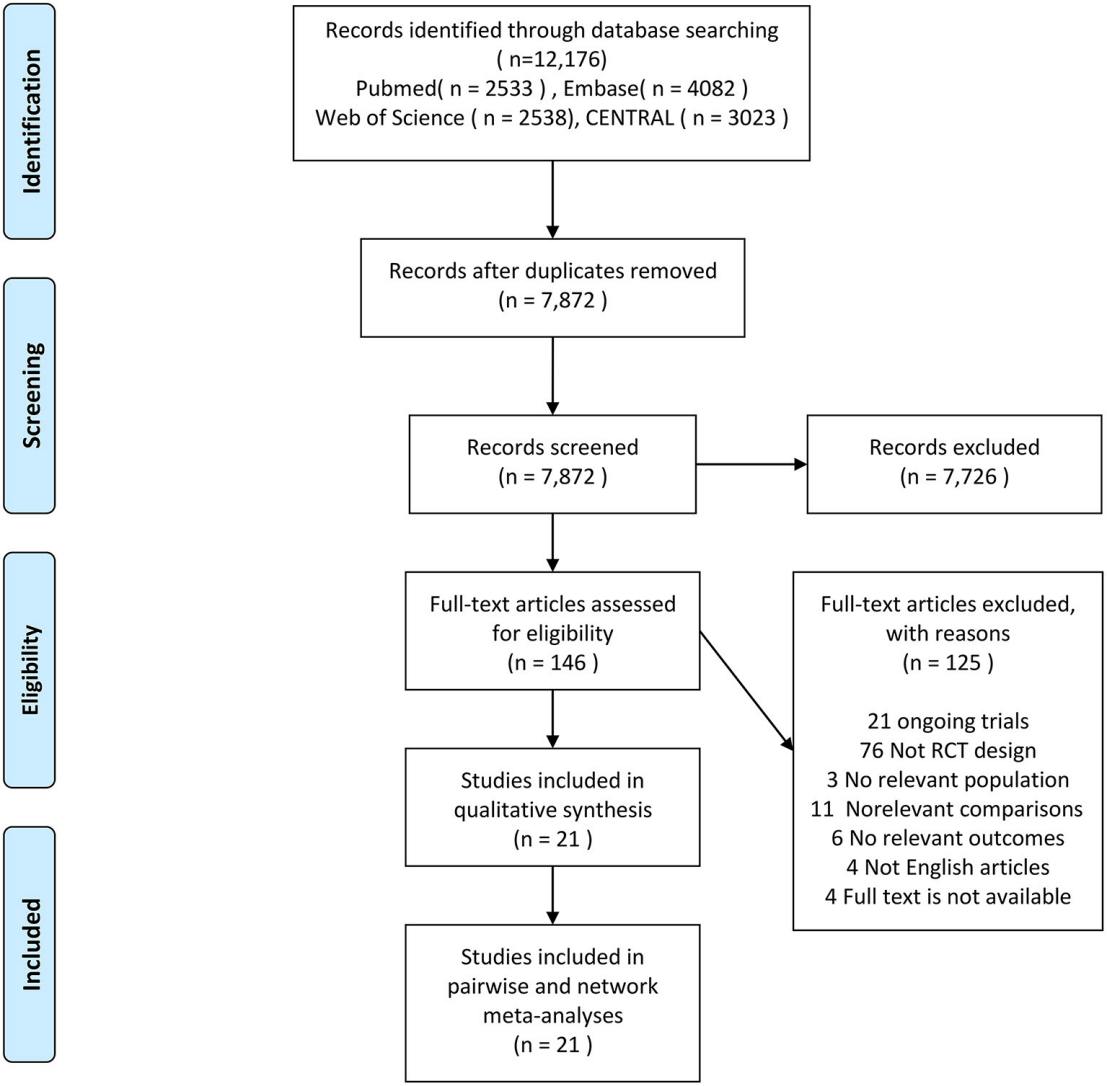
Study selection flowchart. RCT, randomized controlled trial.

**Figure 2:**
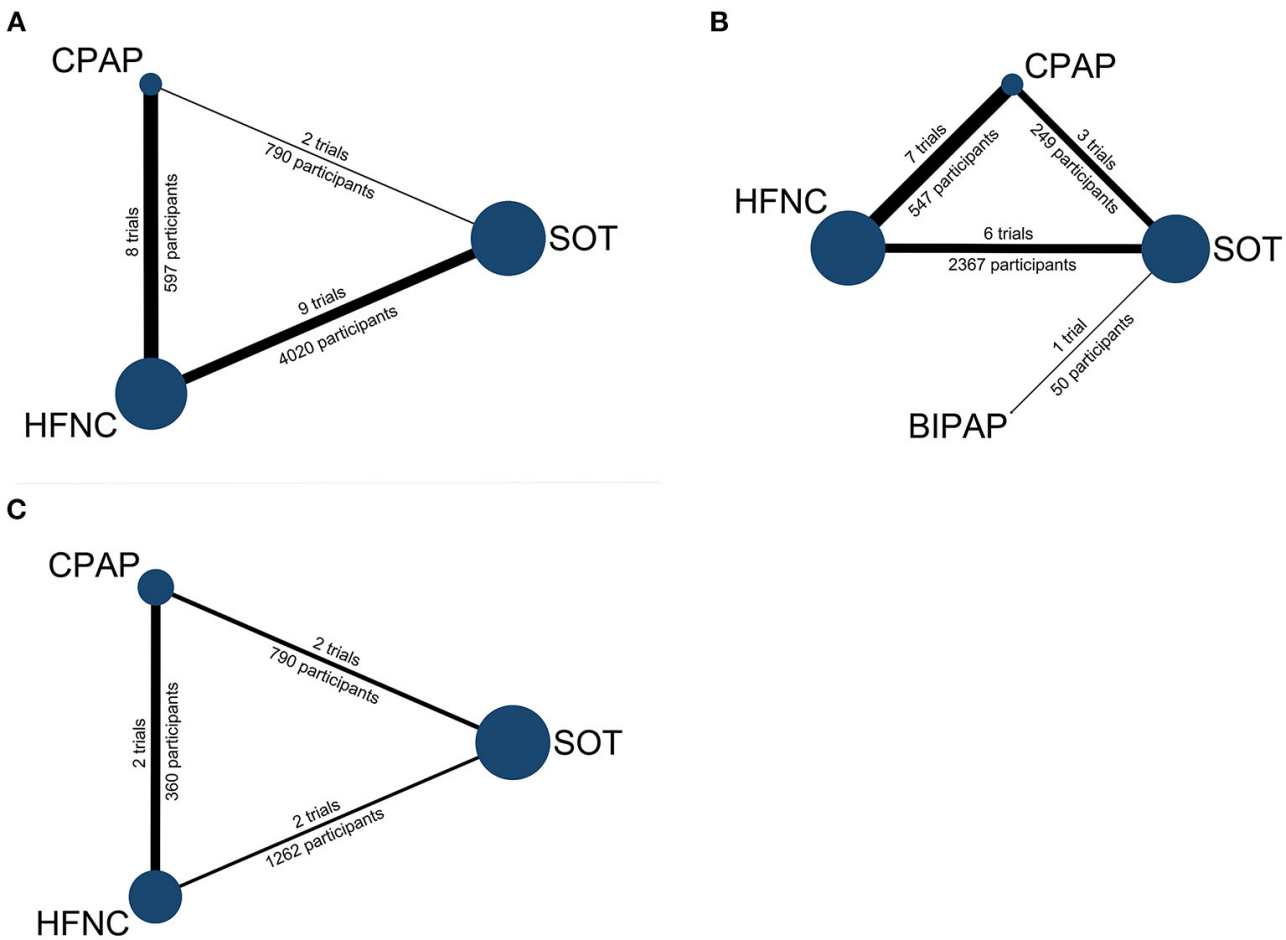
Network geometry: **(A)** treatment failure, **(B)** intubation, **(C)** in-hospital mortality. Node size represents the number of participants and the edge widths are proportional to the number of studies. BIPAP, bilevel positive airway pressure; CPAP, continuous positive airway pressure; HFNC, high-flow nasal cannula; SOT: standard oxygen therapy.

The literature search yielded 12,176 records, and 146 proved potentially eligible. Of these, we excluded 125 studies (see Figure 1) and included 21 trials (11–18, 29–41) with 5,342 children aged 19 days to 16 years. The literature search process is presented in Figure 1 and the network geometry is presented in Figure 2. Descriptive data for included studies are listed in Table 1. Study sample sizes ranged from 28 to 1,472. Among the included trials, three studies (18, 29, 41) compared CPAP with SOT, nine (11–13, 15, 16, 32, 35, 36, 39) compared HFNC with SOT, seven (14, 17, 33, 34, 37, 38, 40) compared HFNC with CPAP, one (30) compared BIPAP with SOT, and one 3-arm study (31) compared CPAP with both HFNC and SOT. Ten studies (47.6%) were conducted in in high-income countries, eight (38.1%) in middle-income countries, and three (14.3%) in low-income countries (42). Twelve studies (12, 13, 16, 17, 29, 32, 33, 35, 36, 38, 40, 41) were conducted in patients with acute bronchiolitis, and the other nine trials (11, 14, 15, 18, 30, 31, 34, 37, 39) were performed in patients with WHO defined severe pneumonia. Treatment failure was determined by clinical signs such as heart rate, respiratory rate, SpO_2_, and a need to escalate treatment. The criteria for treatment failure varied slightly among included studies and are summarized in Supplementary Table 1.

**Table 1:** Characteristics of the studies included in the network meta-analysis.

**Study**	**Age**	***N***	**Setting**	**Inclusion criteria**	**Treatment group**		**Control group**		**Main outcomes**
					**Name**	**Interface**	**Name**	**Interface**	
Chisti et al. (31) Bangladesh	<5 y	225	PICU	Severe pneumonia by WHO definition, and SpO_2_ < 90%	CPAP	Nasal prong	HFNC SOT	Nasal cannula Nasal cannula	Treatment failure, in-hospital mortality, intubation rate
Ergul et al. (35) Turkey	<24 mon	60	PICU	Moderate-to-severe bronchiolitis, and need for supplemental oxygen if SpO_2_ < 92%	HFNC	Nasal cannula	SOT	Mask	Treatment failure
Franklin et al. (15, 36) Australia, New Zealand	<12 mon	1,472	ED, general ward	Bronchiolitis with a need for supplemental oxygen therapy	HFNC	Optiflow junior cannula	SOT	Nasal cannula	Treatment failure, intubation rate
Yañez et al. (30) United Kingdom	1 mon to 15 y	50	PICU	Respiratory failure based on oxygen requirement > 50% for SpO_2_ > 94%, and with moderate to severe respiratory distress	BIPAP	Facial mask	SOT	Mask	Intubation rate
Kepreotes et al. (32) Australia	<24 mon	202	ED	Moderate bronchiolitis by NSW health clinical practice guideline	HFNC	Nasal cannula	SOT	Nasal cannula	Treatment failure, in-hospital mortality
Lal et al. (41) India	1 mon to 1 y	72	General ward	Diagnosis of bronchiolitis defined as respiratory distress with wheezing on auscultation and hyperinflated lung	CPAP	Gregory circuit	SOT	Mask or hood	Intubation rate
McCollum et al. (18) Malawi	1–59 mon	644	General ward	WHO-defined severe pneumonia with one or more high-risk conditions (HIV infection or exposure, severe malnutrition, hypoxemia)	CPAP	Nasal mask or nasal prong	SOT	Nasal prong	Treatment failure, in-hospital mortality
Milési et al. (33) France	1 d to 6 mon	142	PICU	Bronchiolitis, and moderate to severe respiratory distress	HFNC	Optiflow system	CPAP	Infant Flow Ventilator or FlexiTrunk infant interface	Treatment failure, intubation rate
Ramnarayan et al. (37) United Kingdom	36 wk to 16 y	29	PICU	One or more criteria for respiratory support: (1) hypoxia; (2) acute respiratory acidosis; (3) moderate respiratory	HFNC	Helmet, nasal prong, or mask	CPAP	Helmet, nasal prong, or mask	Treatment failure, in-hospital mortality
Sarkar et al. (38) India	28 d to 1 y	31	PICU	Severe bronchiolitis consistent with clinical features, SpO_2_ < 92% in room air, and RDAI ≥ 11	HFNC	Nasal prong	CPAP	Nasal prong or nasal mask	Treatment failure, intubation rate
Sitthikarnkha et al. (39) Thailand General ward	1 mon to 5 y	98	PICU	Respiratory distress with respiratory rate greater than normal, signs of increased work of breathing, or SpO_2_ < 95% in room air	HFNC	Nasal cannula	SOT	Nasal cannula, face mask, or oxygen box	Treatment failure, intubation rate
Thia et al. (29) United Kingdom	<1 y	31	NR	Bronchiolitis and capillary PCO_2_ measurements > 6 kPa	CPAP	Nasal prong	SOT	Nasal prong or face mask	Intubation rate
Vitaliti et al. (34) Italy	1 mon to 2 y	40	ED	Patients with hemodynamically stable hypoxemia	CPAP	Helmet	HFNC	Nasal cannula	Treatment failure, intubation rate
Durand et al. (16) France	7 d to 6 mon	268	ED	Bronchiolitis with SpO_2_ < 95% on room air and m-WCAS score between 2 and 5	HFNC	Optiflow junior infant size cannula	SOT	NR	Treatment failure, intubation rate
Vahlkvist et al. (40) Denmark	<2 y	50	General ward	Bronchiolitis and need for respiratory support	CPAP	Nasal prong	HFNC	Nasal prong	Treatment failure
Liu et al. (14) China	<2 y	84	ED	Mild to moderate respiratory failure due to pneumonia	CPAP	NR	HFNC	NR	Treatment failure, intubation rate
Türe et al. (12) Turkey	<2 y	75	ED	Moderate or severe bronchiolitis	HFNC	Optiflow system	SOT	Face mask	Treatment failure
Maitland et al. (11) Ugandan, Kenyan	28 d to 12 y	1115	General ward	WHO clinical definitions of severe pneumonia plus SpO_2_ < 92%	HFNC	Nasal cannula	SOT	Nasal prong, catheter or mask	Treatment failure, in-hospital mortality
Franklin et al. (15, 36) Australia	1 mon to 16 y	563	ED, general ward	Respiratory failure with oxygen requirement to maintain SpO_2_ ≥ 92%, and admission to hospital	HFNC	OptiflowTM junior 2 nasal interfaces or adult cannula	SOT	Subnasal interface or Hudson mask	Treatment failure, intubation rate
Cesar et al. (17) Brazil	<9 mon	63	PICU	Diagnosis of bronchiolitis of moderate severity or greater	CPAP	Nasal prong	HFNC	Nasal cannula	Treatment failure, intubation rate
Murphy et al. (13) South Africa	1 mon to 2 y	28	High-care area	Diagnosis of bronchiolitis with moderate/severe respiratory distress and hypoxemia (oxygen saturation <92% in room air)	HFNC	Nasal cannula	SOT	Nasal cannula or face mask	Intubation rate

*BIPAP, bilevel positive airway pressure; CPAP, continuous positive airway pressure; d, day; ED, emergency department; HFNC, high-flow nasal cannula; mWCAS, modified Wood's clinical asthma score; NR, not reported; PICU, pediatric intensive care unit; mon, month; RDAI, respiratory distress assessment index; SOT, standard oxygen therapy; SpO_2_, arterial pulse oximetry; WHO, World Health Organization; wk, week; y, year*.

### Risk of Bias Assessment

The risk of bias is summarized in Supplementary Figure 1. Due to the audible and visible differences between the device of oxygen delivery, none of the studies could be blinded for clinicians, researchers or patients. Overall, as none of the trials was at low risk of bias in all domains, we assessed all trials to be at high risk of bias.

### Network Meta-Analysis

The results of pairwise comparisons are shown in Supplementary Figures 2–4.

### Primary Outcomes

#### Treatment Failure

**Figure 3:**
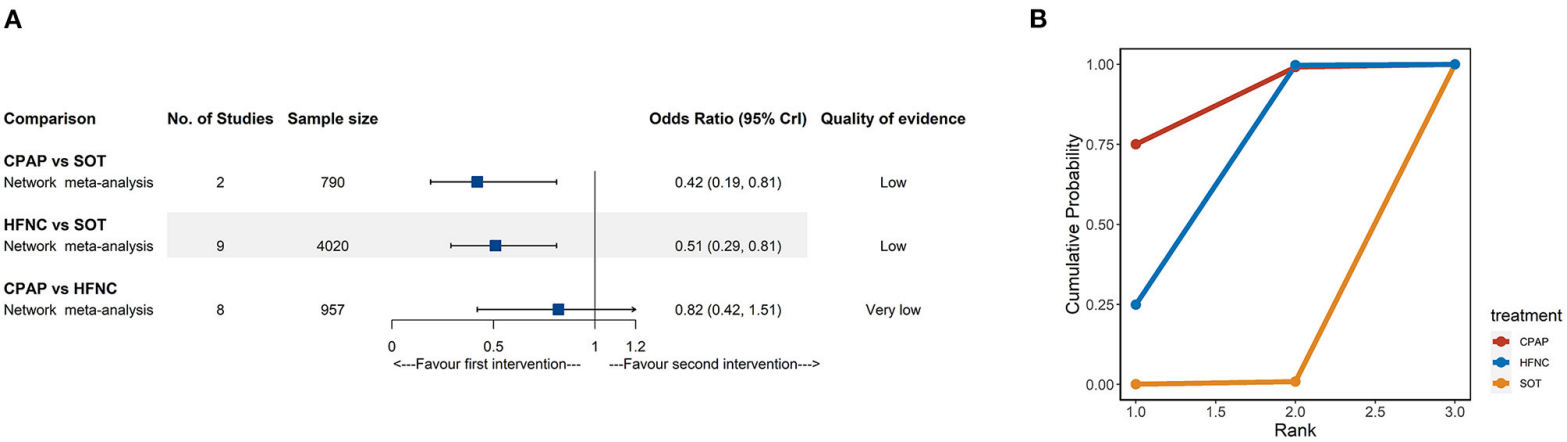
Network meta-analyses results **(A)** and cumulative probability under different rankings **(B)** for treatment failure. CPAP, continuous positive airway pressure; HFNC, high-flow nasal cannula; SOT, standard oxygen therapy.

Seventeen trials (5,182 participants) containing three interventions (SOT, CPAP, and HFNC) provided data on treatment failure. For NMA results (Figure 3A; Supplementary Table 2), both CPAP (OR: 0.42, 95% CrI: 0.19–0.81, low quality) and HFNC (OR: 0.51, 95% CrI: 0.29–0.81, low quality) were associated with lower risk of treatment failure when compared to SOT. The SUCRA for CPAP, HFNC, and SOT were 89.0, 60.5, and 42.1%, respectively (Figure 3B; Supplementary Table 5).

#### Intubation Rate

**Figure 4:**
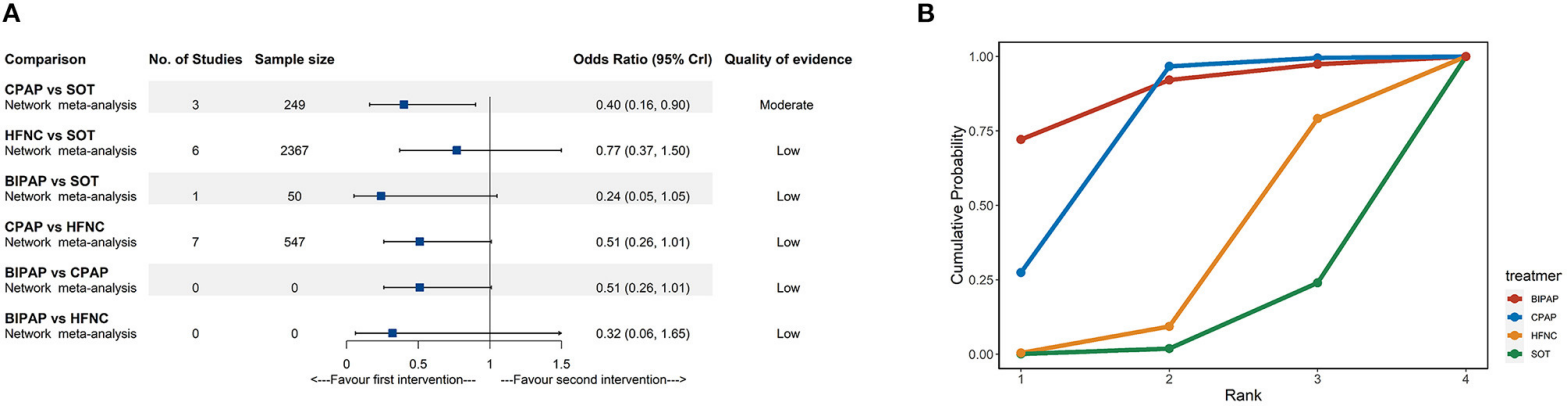
Network meta-analyses results **(A)** and cumulative probability under different rankings **(B)** for intubation. BIPAP, bilevel positive airway pressure; CPAP, continuous positive airway pressure; HFNC, high-flow nasal cannula; SOT, standard oxygen therapy.

Fifteen trials (2,989 participants) comparing four interventions (SOT, CPAP, HFNC, and BIPAP) reported intubation rates. According to the NMA results (Figure 4A; Supplementary Table 3), compared with SOT, CPAP (OR: 0.40, 95% CrI: 0.16–0.90, moderate quality) was associated with a lower risk of intubation. The SUCRA for BIPAP, CPAP, HFNC, and SOT were 88.1, 73.0, 28.7, and 10.2%, respectively (Figure 4B; Supplementary Table 5). The SUCRA results should be interpreted cautiously as the credible intervals were very wide for most comparisons.

### Secondary Outcome

#### In-Hospital Mortality

**Figure 5:**
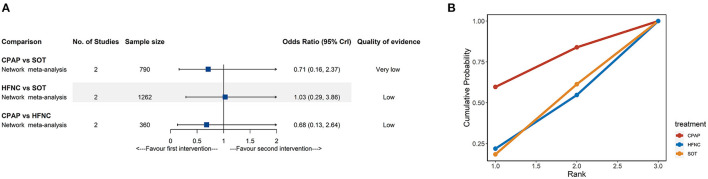
Network meta-analyses results **(A)** and cumulative probability under different rankings **(B)** for in-hospital mortality. CPAP, continuous positive airway pressure; HFNC, high-flow nasal cannula; SOT, standard oxygen therapy.

Deaths were reported in only four studies (11, 18, 31, 37) involving 2,014 participants. As shown in Figure 5A, there were no significant differences for mortality rate among the different treatments from NMA. The network evidence was low or very low quality in all cases (Supplementary Table 4). Similarly, all CrIs were very wide and included the null value; the SUCRA results should be interpreted cautiously (Figure 5B; Supplementary Table 5).

### Network Meta-Regression and Sensitivity Analyses

For treatment failure and intubation rate, the results of network meta-regression demonstrated that there were no statistically significant subgroup effects regarding mean age, country income, mean SpO_2_ on admission, study location, and type of disease. The full details of the subgroup and network meta-regression analyses are reported in Supplementary File (Supplementary Figures 5, 6). We could not perform further analysis for in-hospital mortality due to the limited number of studies.

The McCollum study (18) did not report the intubation rate; therefore, sensitivity analyses were conducted for the other two outcomes (see Supplementary Tables 6, 7). After excluding the McCollum study (18), sensitivity network analysis revealed a lower rate of treatment failure for CPAP (OR: 0.54, 95% CrI: 0.32–0.90), when compared to HFNC. The remaining results were consistent with our primary analyses.

#### Heterogeneity and Inconsistency Analyses

Heterogeneity and inconsistency assessments are detailed in Supplementary Figures 7–9.

For treatment failure, two comparisons (CPAP vs. HFNC and HFNC vs. SOT) were found incoherent in the node-splitting assessment. The heterogeneity analysis suggested high heterogeneity in one comparison (CPAP vs. SOT). As for intubation rate, no evidence of inconsistency or heterogeneity between the studies was found. Regarding in-hospital mortality, there was no evidence for inconsistency. However, the heterogeneity test indicated high heterogeneity in one comparison (CPAP vs. SOT).

### Publication Bias

There was no obvious indication of asymmetry according to the comparison-adjusted funnel plots (Supplementary Figure 10).

## Discussion

In this first network meta-analysis comparing different NIV modalities in children with ALRI, we found that CPAP reduced the risk of intubation when compared to standard oxygen therapy. What is more, a lower incidence of treatment failure rate was found for both CPAP and HFNC when compared to standard oxygen therapy. However, there was no evidence of differences for all modalities concerning in-hospital mortality. Overall, the certainty of evidence was low or very low for all the outcomes, not only because of the small number of trials in each node, but also due to the variability of methodology and participants among studies.

A pairwise meta-analysis (10) of different NIV modalities in children with ALRI by Luo and colleagues showed that HFNC reduced treatment failure than SOT, despite no reduction in intubation rate. They also reported that HFNC had a higher risk of treatment failure when compared with CPAP. Our primary and sensitivity analyses are consistent with these previous findings. However, we found that CPAP use was associated with lower risks of treatment failure and intubation compared to SOT. This contrasts with the previous study by Luo et al. (10) where no significant differences between these two interventions were observed. The differences between their results and ours may be explained by the following reasons. First, this NMA had included more recently published studies. Second, this was an NMA where apart from the direct synthesis, the indirect evidence also contributed to the overall effect estimate.

Compared with low-flow oxygen therapy, CPAP works by delivering continuous distending pressure (43). Application of CPAP prevents collapse of alveoli and small airways during expiration, thus increases functional residual capacity (43, 44). HFNC has been shown to improve alveolar ventilation and carbon dioxide elimination (45) by decreasing the dead space through establishing washout in the nasopharyngeal space (46). High flow rates can also provide variable end-expiratory distending pressure based on flow rate (47, 48). Such mechanisms may explain the greater benefit of CPAP and HFNC over standard oxygen therapy in some patients. However, the airway pressure generated by HFNC is determined not only by the HFNC flow, but also by the degree of air leak from both mouth and nose (44). This may partially explain why HFNC displayed a less favorable clinical response than CPAP.

In comparison to CPAP, BIPAP delivers positive airway pressure at two different levels during inspiration and expiration, and could more efficiently decrease inspiratory work of breathing than CPAP (49). However, our pooled results suggested that when compared with SOT, BIPAP did not decrease risk of intubation. It should be noted that most of the evidence that contributed to this comparison was indirect. There was only one trial included in this meta-analysis that directly assessed the effect of BIPAP by comparing it with SOT, and this study did not report treatment failure or mortality. Therefore, conclusions regarding the relative effectiveness of BIPAP are limited, and further research is required.

In our study, we were unable to show benefits among different interventions on in-hospital mortality. Several possible explanations may account for these results. First, among the enrolled studies, 12 (57%) were focused on patients with acute bronchiolitis. Given that very low incidence of mortality has been reported since the introduction of NIV for acute bronchiolitis (33), this would make it difficult to detect differences among various methods of respiratory support. Second, in several trials (32, 33, 36–38), once treatment failure or deterioration occurred, crossover to an alternative respiratory support as rescue therapy was allowed, and the crossover may have avoided exacerbation of respiratory distress. The dilution effect of crossover may partially explain the absence of differences between any of the interventions on mortality.

Among the studies we included, 10 (47.6%) were conducted in high-income countries, where 60%−81% of children improved with standard oxygen therapy alone. Considering that non-invasive oxygen approaches are more costly and complex than standard oxygen therapy, these strategies might not be needed as preferred forms of treatment for mild cases. In resource-rich regions, more research is needed to identify children who are likely to deteriorate on standard oxygen therapy, and therefore who would benefit from the early initiation of non-invasive ventilation. However, at the other end of the spectrum of clinical severity, several included studies (11, 18, 31) from low- and middle-income countries (LMICs) showed case fatality rates of 7–14%. Meta-regression and subgroup analyses of country income (LMICs vs. high-income) found that there were no significant interactions between subgroups with regard to treatment failure and intubation rates. However, due to the low number of studies that reported mortality, subgroup analysis for in-hospital mortality was not conducted. Existing data are scarce on the effect of non-invasive ventilation on mortality of children in LMICs, and the results of previous studies remain controversial (11, 18, 31, 50). More studies will be necessary to define the role of various non-invasive ventilation modalities in resource-limited settings.

Some limitations of this review should be noted. First, there was limited statistical power for some comparisons, such as intubation rate and in-hospital mortality, because of the small numbers of trials forming several of the nodes in this network of the meta-analysis. Notably, only one of the included studies examined the efficacy of BIPAP, indicating that further studies are necessary to obtain more precise effects estimates. Second, this study involved a heterogeneous population with a wide age range (0–18 years) and disease spectrum (bronchiolitis and pneumonia). However, the heterogeneity of the study population may enhance the generalizability of the results. Moreover, to address this problem, multiple subgroup analyses were performed. Multiple subgroup results remained consistent with the overall findings. Hence, we believed that the results of our study were credible. Third, the included trials used variable definitions of treatment failure, which might produce heterogeneity. Finally, when eliminating the McCollum study (18) in the sensitivity analysis, some non-significant findings became significant, which encourages further studies to validate these results.

## Conclusions

Compared to standard oxygen therapy, the use of CPAP reduced intubation rate in children with ALRI and the need for supplemental oxygen. Additionally, both CPAP and HFNC decreased the risk of treatment failure when compared with standard oxygen therapy. However, evidence is still lacking to show benefits concerning mortality between different interventions. Further large-scale, multicenter studies are needed to confirm our results.

## Data Availability Statement

The original contributions presented in the study are included in the article/Supplementary Material, further inquiries can be directed to the corresponding author.

## Author Contributions

ZW is responsible for the conception, data search, data analysis, statistical analyses, data interpretation, writing, and revising the manuscript. YH is responsible for inclusion and exclusion of studies, assessment of methodological quality, data extraction, writing, and revising the manuscript. XZ is responsible for inclusion and exclusion of studies, assessment of methodological quality, data extraction, and statistical analyses. ZL is responsible for the supervision and reviewed the manuscript for important intellectual content. All authors approved the manuscript.

## Conflict of Interest

The authors declare that the research was conducted in the absence of any commercial or financial relationships that could be construed as a potential conflict of interest.

## Publisher's Note

All claims expressed in this article are solely those of the authors and do not necessarily represent those of their affiliated organizations, or those of the publisher, the editors and the reviewers. Any product that may be evaluated in this article, or claim that may be made by its manufacturer, is not guaranteed or endorsed by the publisher.
