# Supplementary material for: Non-Invasive Ventilation Strategies in Children With Acute Lower Respiratory Infection: A Systematic Review and Bayesian Network Meta-Analysis
Source: Front Pediatr. 2021 Dec 2;9:749975. doi: 10.3389/fped.2021.749975 (PMC8677331; doi:10.3389/fped.2021.749975)

Supplementary Material

# Full search strategy for Pubmed database.

#1 Positive-Pressure Respiration[MeSH Terms]

#2 “high-flow nasal cannul*”[Text Word] OR “high flow cannul*”[Text Word] OR “high flow oxygen therapy”[Text Word] OR “high flow oxygen”[Text Word] OR “high flow therapy”[Text Word] OR “HFNC”[Text Word] OR “high flow high humidity nasal cannul*”[Text Word] OR “high-flow warm humidified oxygen” [Text Word] OR “hot humidified high-flow”[Text Word] OR NPPV[Text Word] OR NIPPV[Text Word] OR CPAP[Text Word] OR bCPAP[Text Word] OR "bubble CPAP"[Text Word] OR nCPAP[Text Word] OR BiPAP[Text Word] OR non-invasive[Text Word] OR "non invasive"[Text Word] OR positive-pressure[Text Word] OR "positive pressure"[Text Word] OR "pressure support"[Text Word] OR pressure-support[Text Word] OR "positive airway"[Text Word] OR positive-airway[Text Word] OR airway-pressure[Text Word] OR "airway pressure"[Text Word] OR "pressure control"[Text Word] OR pressure-control[Text Word] OR bi-level*[Text Word] OR bilevel*[Text Word] OR "ventilat* support*"[Text Word] OR "volume control"[Text Word] OR volume-control[Text Word] OR "nasal* ventilat*"[Text Word] OR "mechanical* ventilat*"[Text Word] OR “oxygen therapy” [Text Word] OR “oxygen supplementation” [Text Word] OR “oxygen delivery” [Text Word] OR “conventional oxygen therapy” [Text Word] OR COT[Text Word]

#3 #1 or #2

#4 Pneumonia[MeSH Terms]

#5 Bronchitis[MeSH Terms]

#6 Respiratory Tract Infections[MeSH Terms]

#7 Bronchiolitis[MeSH Terms]

#8 Respiratory Syncytial Viruses[MeSH Terms]

#9 respirat*[Text Word] OR pulmonary*[Text Word] OR "respiratory distress"[Text Word] OR "respiratory insufficiency"[Text Word] OR pneumon*[Text Word] OR bronchopneumon*[Text Word] OR pleuropneumon*[Text Word] OR bronchit*[Text Word] OR tracheobronchit*[Text Word] OR respiratory Sounds[Text Word] OR wheez*[Text Word] OR respiratory tract infection*[Text Word] OR acute respiratory infection*[Text Word] OR lower respiratory infection*[Text Word] OR lower respiratory tract infection*[Text Word] OR lrti[Text Word] OR bronchiolit*[Text Word] OR respiratory syncytial virus infections[Text Word] OR respiratory syncytial virus*[Text Word] OR rsv[Text Word]

#10 #4 or #5 or #6 or #7 or #8 or #9

#11 schoolchild*[Text Word] OR schoolchild[Text Word] OR school child[Text Word] OR school child*[Text Word] OR kid[Text Word] OR kids[Text Word] OR toddler*[Text Word] OR adolescent[Text Word] OR adoles*[Text Word] OR teen*[Text Word] OR boy[Text Word] OR girl*[Text Word] OR minors[Text Word] OR minors*[Text Word] OR underag*[Text Word] OR under ag*[Text Word] OR juvenil*[Text Word] OR youth*[Text Word] OR kindergar*[Text Word] OR puberty[Text Word] OR puber*[Text Word] OR pubescen*[Text Word] OR prepubescen*[Text Word] OR prepuberty*[Text Word] OR pediatrics[Text Word] OR pediatric*[Text Word] OR paediatric*[Text Word] OR peadiatric*[Text Word] OR schools[Text Word] OR nursery school*[Text Word] OR preschool*[Text Word] OR pre school*[Text Word] OR primary school*[Text Word] OR secondary school*[Text Word] OR elementary school*[Text Word] OR elementary school[Text Word] OR high school*[Text Word] OR highschool*[Text Word] OR school age[Text Word] OR schoolage[Text Word] OR school age*[Text Word] OR schoolage*[Text Word] OR infancy[Text Word]

#12 randomized controlled trial[Publication Type] OR controlled clinical trial[Publication Type] OR randomized[Title/Abstract] OR placebo[Title/Abstract] OR randomly[Title/Abstract] OR clinical trials as topic[MeSH Major Topic] OR trial[Publication Type]

#13 animals[MeSH Terms] NOT humans[MeSH Terms]

#14 #12 NOT #13

#15 #3 AND #10 AND #11 AND #14

# Supplementary Table 1. A summary of treatment failure definition in all included studies.

| **Features**  **Studies** | **Intervention** | **Oxygenation** | **Respiratory rate** | **Heart rate** | **Arterial blood gas** | **Respiratory distress** | **Timing** | **Others** |
| --- | --- | --- | --- | --- | --- | --- | --- | --- |
| Chisti 2015 | CPAP vs SOT and HFNC | Severe hypoxemia  (SpO_2_ < 85%) | - | - | PaCO_2_ > 60 mmHg and pH < 7.2 in capillary blood gas | Signs of respiratory distress including moderate to severe chest wall in-drawing, tracheal tug, nasal flaring, or grunting respirations | ≥30 min of study intervention | Need for mechanical ventilation; death at any time during hospital stay or within 30 d of discharge; or left hospital against medical advice |
| Ergul 2018 | HFNC vs SOT | Persistence of low SpO_2_ < 92% in the HFNC group/oxygen flow rate of 15L/min in the SOT group | No change or an increase in respiration rate | No change or an increase in heart rate | - | - | At any time point during hospital stay | - |
| Franklin 2018 | HFNC vs SOT | FIO_2_ ≥0.4 in HFNC group; supplemental oxygen > 2L/min in SOT group to maintain SpO_2_ ≥92% or 94% depending on the institution | Unchanged or increased | Unchanged or increased | - | - | At the point of hospital admission | An escalation of care during hospital admission; hospital internal early-warning tool triggered a medical review |
| Kepreotes 2017 | HFNC vs SOT | SpO2 < 90% on maximum therapy | Red zone on an age-appropriate SPOC for respiratory rate on maximum therapy | Red zone on an age-appropriate SPOC for heart rate on maximum therapy | - | Red zone on an age-appropriate SPOC for respiratory distress score (severe) on maximum therapy | - | Clinical decision by the treating physician or medical delegate that the current treatment was insufficient to reverse the deterioration |
| McCollum 2019 | CPAP vs SOT | Continued oxygen or CPAP requirement | Any respiratory  danger sign | - | - | - | Day 14 of hospitalisation | Temperature ≥ 38 °C |
| Mile´si 2017 | CPAP vs HFNC | - | Increase > 10bpm compared with baseline, with respiratory rate > 60bpm | - | - | - | - | More than 2 severe apnea episodes per hour; 1-point increase in mWCAS compared with baseline;  1-point increase in the EDIN score compared with baseline, with EDIN > 4 |
| Ramnarayan 2018 | CPAP vs HFNC | - | - | - | - | - | - | An escalation of care or a crossover use of respiratory support |
| Sarkar 2018 | CPAP vs HFNC | FIO_2_ > 0.6 for HFNC with maximum oxygen flow rate to maintain SpO_2_ > 94%; FIO2 > 0.6 for nCPAP with PEEP > 8 cmH2O | Unchanged or increased | Unchanged or increased | - | No improvement or increase in RDAI score | - | - |
| Sitthikarnkha 2018 | HFNC vs SOT | FIO_2_ > 0.5 | NOT reduced by 20% or NOT within normal range | NOT reduced by 20% or NOT within normal range | - | - | - | - |
| Vitaliti 2017 | CPAP vs HFNC | - | - | - | - | - | - | - |
| Durand 2020 | HFNC vs SOT | FiO_2_ > 40% (HFNC group) or flow oxygen > 2 L/min (control group) for SpO_2_ ≥ 94% | - | - | Increased PaCO_2_ compared to  baseline and/or above 60 mmHg at hour 6 | Elevated m-WCAS score (≥ 1 point) at hour 6 compared to baseline and/or any scores higher than 5 | - | Refractory apnea episodes (more than 3 per hour) |
| Vahlkvist 2020 | CPAP vs HFNC | - | - | - | - | - | - | A switch of system  (CPAP to HFNC/HFNC to CPAP) or to transmission to the PICU |
| Liu 2020 | CPAP vs HFNC | - | - | - | - | - |  | Need for transmission to the PICU and mechanical ventilation |
| Türe 2020 | HFNC vs SOT | Failure to return back to normal | Failure to return back to normal | Failure to return back to normal | - | - | 24 hours after admission | - |
| Maitland 2021 | HFNC vs SOT | Persistent hypoxaemia SpO_2_ < 92% | - | - | - | Persistent respiratory distress | 48 hours after admission | - |
| Franklin 2020 | HFNC vs SOT | FIO_2_ ≥0.4 in HFNC group; supplemental oxygen > 2L/min (subnasal) or > 8 L/min (Hudson mask) in SOT group to maintain SpO_2_ ≥92% | Unchanged or increased | Unchanged or increased | - | - | - | Hospital early warning tool necessitates a medical review and the clinician decided on an escalation of care |
| Cesar 2020 | CPAP vs HFNC | - | - | - | - | - | - | The need to escalate support to noninvasive bilevel pressure ventilation, or endotracheal intubation |

CPAP: continuous positive airway pressure; EDIN, neonatal pain and discomfort scale; HFNC, humidified high-flow nasal cannula; mWCAS: modified Wood’s clinical asthma score; PICU: pediatric intensive care unit, SOT, standard oxygen therapy; SpO_2_: arterial pulse oximetry; SPOC, standard pediatric observation charts; RDAI: respiratory distress assessment index

# Supplementary Table 2. The confidence in OR for treatment failure by GRADE system*.

| Comparison | Study limitations^a^ | Imprecision^b^ | Heterogeneity and  Inconsistency^c^ | Indirectness^d^ | Publication bias^e^ | Confidence in OR  for mortality rate |
| --- | --- | --- | --- | --- | --- | --- |
| CPAP *vs.* SOT | 100% of the estimate  from studies at high risk | OR 0.42, 95% CrI 0.19 to 0.81 | High heterogeneity according  to *I^2^* (91%) and *P*-value  (0.001) in direct comparisons.  No inconsistency between the  direct and indirect estimate (Node-split *P*=0.1701). | The treatment effects were not significantly  influenced by clinical  modifiers in the network  meta-regression. | Undetectable by the routine  method. The comparison-  adjusted funnel plot for the  network is not suggestive of any dominant publication bias. | **Low** (Downgrade  by two levels due to  study limitations and heterogeneity). |
| HFNC *vs.* SOT | 100% of the estimate  from studies at high risk | OR 0.51, 95% CrI 0.29 to 0.81 | Mild heterogeneity according  to *I^2^* (36%) and *P*-value  (0.13) in direct comparisons.  Significant inconsistency between the direct and indirect estimate (Node-split *P*=0.0086). | The treatment effects were not significantly  influenced by clinical  modifiers in the network  meta-regression. | Undetectable by the routine  method. The comparison-  adjusted funnel plot for the  network is not suggestive of any dominant publication bias. | **Low** (Downgrade  by two levels due to  study limitations, and  inconsistency). |
| CPAP *vs.* HFNC | 100% of the estimate  from studies at high risk | OR 0.82, 95% CrI 0.42 to 1.51 | Mild heterogeneity according  to *I^2^* (0%) and *P*-value  (0.96) in direct comparisons.  Significant inconsistency between the direct and indirect estimate (Node-split *P*=0.0080). | The treatment effects were not significantly  influenced by clinical  modifiers in the network  meta-regression. | Undetectable by the routine  method. The comparison-  adjusted funnel plot for the  network is not suggestive of any dominant publication bias. | **Very low** (Downgrade  by three levels due to  study limitations, imprecision,and  inconsistency). |

* Salanti G, Del Giovane C, Chaimani A, Caldwell DM, Higgins JP. Evaluating the quality of evidence from a network meta-analysis. PLoS One. 2014 Jul 3;9(7):e99682. ^a^ The consideration for study limitations was based on the relative contributions of risk of bias in direct evidence. We downgraded by one level for high risk of bias. ^b^ The consideration for imprecision was based on the width of the confidence interval. ^c^ The consideration for heterogeneity and inconsistency was based on the corresponding *P* value. We downgraded by one level for significant heterogeneity and inconsistency, respectively. ^d^ The consideration for indirectness was based on the results of network meta-regression. We downgraded by one level if the confidence interval of correlation coefficient not included 0. ^e^ The consideration for publication bias was based on the comparison-adjusted funnel plot.

Crls: Cridible intervals, ORs: odd ratios, CPAP: continuous positive airway pressure, HFNC: high-flow nasal cannula, SOT: standard oxygen therapy

# Supplementary Table 3. The confidence in OR for intubation rate by GRADE system*.

| Comparison | Study limitations^a^ | Imprecision^b^ | Heterogeneity and  Inconsistency^c^ | Indirectness^d^ | Publication bias^e^ | Confidence in OR  for mortality rate |
| --- | --- | --- | --- | --- | --- | --- |
| CPAP *vs.* SOT | 100% of the estimate  from studies at high risk | OR 0.40, 95% CrI 0.16 to 0.90 | Mild heterogeneity according  to *I^2^* (0%) and *P*-value  (0.41) in direct comparisons.  No inconsistency between the  direct and indirect estimate (Node-split *P*=0.7756. | The treatment effects were not significantly  influenced by clinical  modifiers in the network  meta-regression. | Undetectable by the routine  method. The comparison-  adjusted funnel plot for the  network is not suggestive of any dominant publication bias. | **Moderate** (Downgrade  by one level due to  study limitations). |
| HFNC *vs.* SOT | 100% of the estimate  from studies at high risk | OR 0.77, 95% CrI 0.37 to 1.50 | Mild heterogeneity according  to *I^2^* (6%) and *P*-value  (0.37) in direct comparisons.  No inconsistency between the  direct and indirect estimate (Node-split *P*=0.4699). | The treatment effects were not significantly  influenced by clinical  modifiers in the network  meta-regression. | Undetectable by the routine  method. The comparison-  adjusted funnel plot for the  network is not suggestive of any dominant publication bias. | **Low** (Downgrade  By two levels due to  study limitations, and  imprecision). |
| BIPAP *vs.* SOT | 100% of the estimate  from studies at high risk | OR 0.24, 95% CrI 0.05 to 1.05 | Only one head-to-head study,  and no heterogeneity. No indirect comparison and no node-splitting inconsistency. | Meta-regression not performend due to limited studies. No potential effect modifiers from meta-regression. | Undetectable by the routine  method. The comparison-  adjusted funnel plot for the  network is not suggestive of any dominant publication bias. | **Low** (Downgrade  by one level due to  study limitations, and  imprecision). |
| CPAP *vs.* HFNC | 100% of the estimate  from studies at high risk | OR 0.51, 95% CrI 0.26 to 1.01 | Mild heterogeneity according  to *I^2^* (0%) and *P*-value  (0.99) in direct comparisons.  No inconsistency between the  direct and indirect estimate (Node-split *P*=0.4485). | The treatment effects were not significantly  influenced by clinical  modifiers in the network  meta-regression. | Undetectable by the routine  method. The comparison-  adjusted funnel plot for the  network is not suggestive of any dominant publication bias. | **Low** (Downgrade  by one level due to  study limitations, and  imprecision). |
| BIPAP *vs.* CPAP | 100% of the estimate  from studies at high risk | OR 0.51, 95% CrI 0.26 to 1.01 | No head-to-head study and no  heterogeneity.  Only indirect comparison, and  no node-splitting inconsistency. | Meta-regression not performend due to limited studies. No potential effect modifiers from metaregression. | Undetectable by the routine  method. The comparison-  adjusted funnel plot for the  network is not suggestive of any dominant publication bias. | **Low** (Downgrade  by two levels due to  study limitations, and  imprecision). |
| BIPAP *vs.* HFNC | 100% of the estimate  from studies at high risk | OR 0.32, 95% CrI 0.06 to 1.65 | No head-to-head study and no  heterogeneity.  Only indirect comparison, and  no node-splitting inconsistency. | Meta-regression not performend due to limited studies. No potential effect modifiers from metaregression. | Undetectable by the routine  method. The comparison-  adjusted funnel plot for the  network is not suggestive of any dominant publication bias. | **Low** (Downgrade  by two levels due to  study limitations, and  imprecision). |

* Salanti G, Del Giovane C, Chaimani A, Caldwell DM, Higgins JP. Evaluating the quality of evidence from a network meta-analysis. PLoS One. 2014 Jul 3;9(7):e99682. ^a^ The consideration for study limitations was based on the relative contributions of risk of bias in direct evidence. We downgraded by one level for high risk of bias. ^b^ The consideration for imprecision was based on the width of the confidence interval. ^c^ The consideration for heterogeneity and inconsistency was based on the corresponding *P* value. We downgraded by one level for significant heterogeneity and inconsistency, respectively. ^d^ The consideration for indirectness was based on the results of network metaregression. We downgraded by one level if the confidence interval of correlation coefficient not included 0. ^e^ The consideration for publication bias was based on the comparison-adjusted funnel plot.

BIPAP:bilevel positive airway pressure, Crls: Cridible intervals, ORs: odd ratios, CPAP: continuous positive airway pressure, HFNC: high-flow nasal cannula, SOT: standard oxygen therapy

# Supplementary Table 4. The confidence in OR for mortality rate by GRADE system*.

| Comparison | Study limitations^a^ | Imprecision^b^ | Heterogeneity and  Inconsistency^c^ | Indirectness^d^ | Publication bias^e^ | Confidence in OR  for mortality rate |
| --- | --- | --- | --- | --- | --- | --- |
| CPAP *vs.* SOT | 100% of the estimate  from studies at high risk | OR 0.71, 95% CrI 0.16 to 2.37 | High heterogeneity according  to *I^2^* (87%) and *P*-value  (0.006) in direct comparisons.  No inconsistency between the  direct and indirect estimate (Node-split *P*=0.7385). | The treatment effects were not significantly  influenced by clinical  modifiers in the network  meta-regression. | Comparison-adjusted funnel plot was not conducted to as too few studies. No publication bias from comparison-adjusted funnel plot. | **Very low** (Downgrade  by three levels due to  study limitations,  imprecision, and  heterogeneity). |
| HFNC *vs.* SOT | 100% of the estimate  from studies at high risk | OR 1.03, 95% CrI 0.29 to 3.86 | Mild heterogeneity according  to *I^2^* (0%) and *P*-value  (0.92) in direct comparisons.  No inconsistency between the  direct and indirect estimate (Node-split *P*=0.3880). | The treatment effects were not significantly  influenced by clinical  modifiers in the network  meta-regression. | Comparison-adjusted funnel plot was not conducted to as too few studies. No publication bias from comparison-adjusted funnel plot. | **Low** (Downgrade  by two levels due to  study limitations, and imprecision). |
| CPAP *vs.* HFNC | 100% of the estimate  from studies at high risk | OR 0.68, 95% CrI 0.13 to 2.64 | Low heterogeneity according  to *I^2^* (0%) and *P*-value  (0.60) in direct comparisons.  No inconsistency between the  direct and indirect estimate (Node-split *P*=0.1509). | The treatment effects were not significantly  influenced by clinical  modifiers in the network  meta-regression. | Comparison-adjusted funnel plot was not conducted to as too few studies. No publication bias from comparison-adjusted funnel plot. | **Low** (Downgrade  by two levels due to  study limitations, and imprecision). |

* Salanti G, Del Giovane C, Chaimani A, Caldwell DM, Higgins JP. Evaluating the quality of evidence from a network meta-analysis. PLoS One. 2014 Jul 3;9(7):e99682. ^a^ The consideration for study limitations was based on the relative contributions of risk of bias in direct evidence. We downgraded by one level for high risk of bias. ^b^ The consideration for imprecision was based on the width of the confidence interval. ^c^ The consideration for heterogeneity and inconsistency was based on the corresponding *P* value. We downgraded by one level for significant heterogeneity and inconsistency, respectively. ^d^ The consideration for indirectness was based on the results of network metaregression. We downgraded by one level if the confidence interval of correlation coefficient not included 0. ^e^ The consideration for publication bias was based on the comparison-adjusted funnel plot.

Crls: Cridible intervals, ORs: odd ratios, CPAP: continuous positive airway pressure, HFNC: high-flow nasal cannula, SOT: standard oxygen therapy

# Supplementary Table 5. The SUCRA results for each outcome.

| **Outcome**  **SUCRA^*^(%)**  **Treatments** | Treatment failure | Intubation | Mortality |
| --- | --- | --- | --- |
| BIPAP | NA | 88.1 % | NA |
| CPAP | 89.0 % | 73.0 % | 69.9 % |
| HFNC | 60.5 % | 28.7 % | 39.3 % |
| SOT | 42.1 % | 10.2 % | 40.8 % |

^*^Larger SUCRAs denote more effective interventions. BIPAP:bilevel positive airway pressure, CPAP: continuous positive airway pressure, HFNC: high-flow nasal cannula, SOT: standard oxygen therapy, SUCRA: surface under the cumulative ranking curve

# Supplementary Table 6. Sensitivity network meta-analysis for treatment failure by omitting the trial conducted by McCollum et al. (18) (ORs and 95% Crls).

| Standard Oxygen | **0.26 (0.14, 0.45)** | **0.48 (0.33, 0.64)** |
| --- | --- | --- |
| **3.88 (2.21, 7.22)** | CPAP | **1.87 (1.11, 3.11)** |
| **2.07 (1.55, 3.05)** | **0.54 (0.32, 0.9)** | HFNC |

CPAP: continuous positive airway pressure, Crls: Cridible intervals, HFNC: high-flow nasal cannula, ORs: odd ratios, SOT: standard oxygen therapy

# Supplementary Table 7. Sensitivity network meta-analysis for mortality by omitting the trial conducted by McCollum et al. (18) (ORs and 95% Crls).

| SOT | 0.23 (0.04, 1.08) | 0.78 (0.27, 2.16) |
| --- | --- | --- |
| 4.28 (0.93, 23.15) | CPAP | 3.33 (0.79, 16.63) |
| 1.28 (0.46, 3.64) | 0.3 (0.06, 1.27) | HFNC |

CPAP: continuous positive airway pressure, Crls: Cridible intervals, HFNC: high-flow nasal cannula, ORs: odd ratios, SOT: standard oxygen therapy

# Supplementary Figure 1. Risk of bias assessment of the included studies.


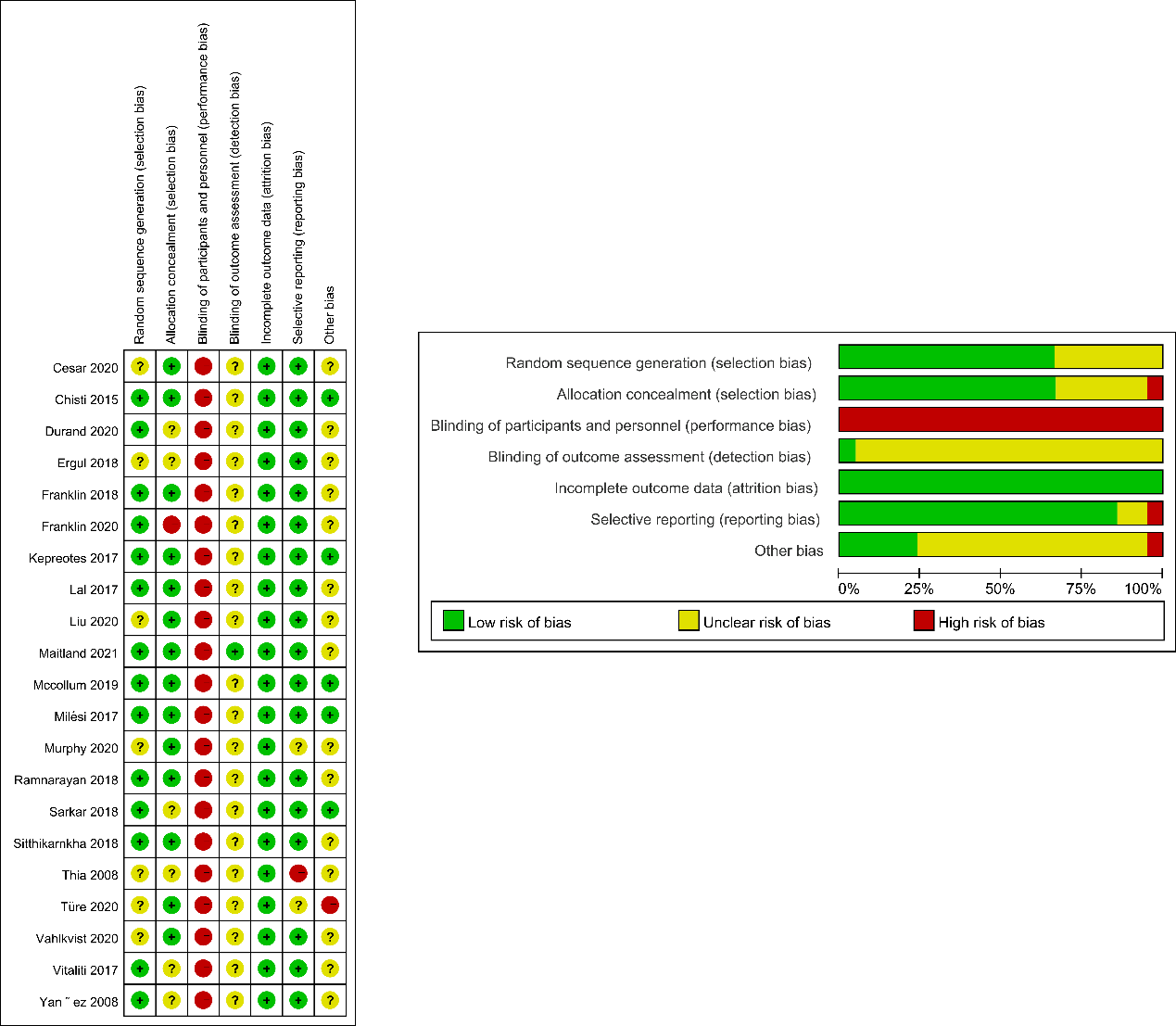


# Supplementary Figure 2. Forest plots for the pairwise comparison of treatment failure. (a) CPAP vs. SOT. (b) HFNC vs. SOT. (c) CPAP vs. HFNC. CPAP: continuous positive airway pressure, HFNC: high-flow nasal cannula, SOT: standard oxygen therapy
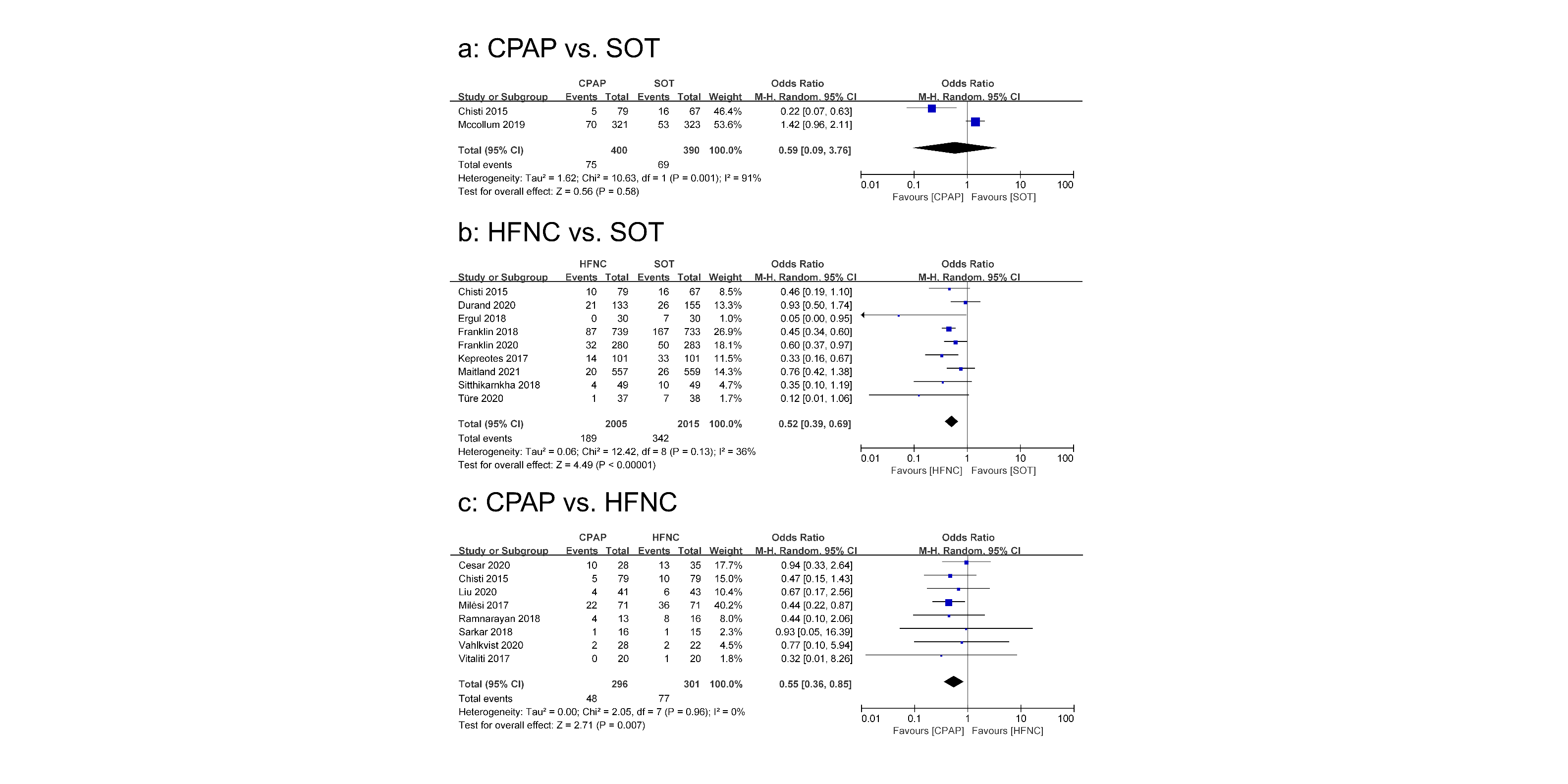


# Supplementary Figure 3. Forest plots for the pairwise comparison of Intubation rate. (a) CPAP vs. SOT. (b) HFNC vs. SOT. (c) CPAP vs. HFNC. (d) BIPAP vs. SOT. BIPAP: bilevel positive airway pressure, CPAP: continuous positive airway pressure, HFNC: high-flow nasal cannula, SOT: standard oxygen therapy
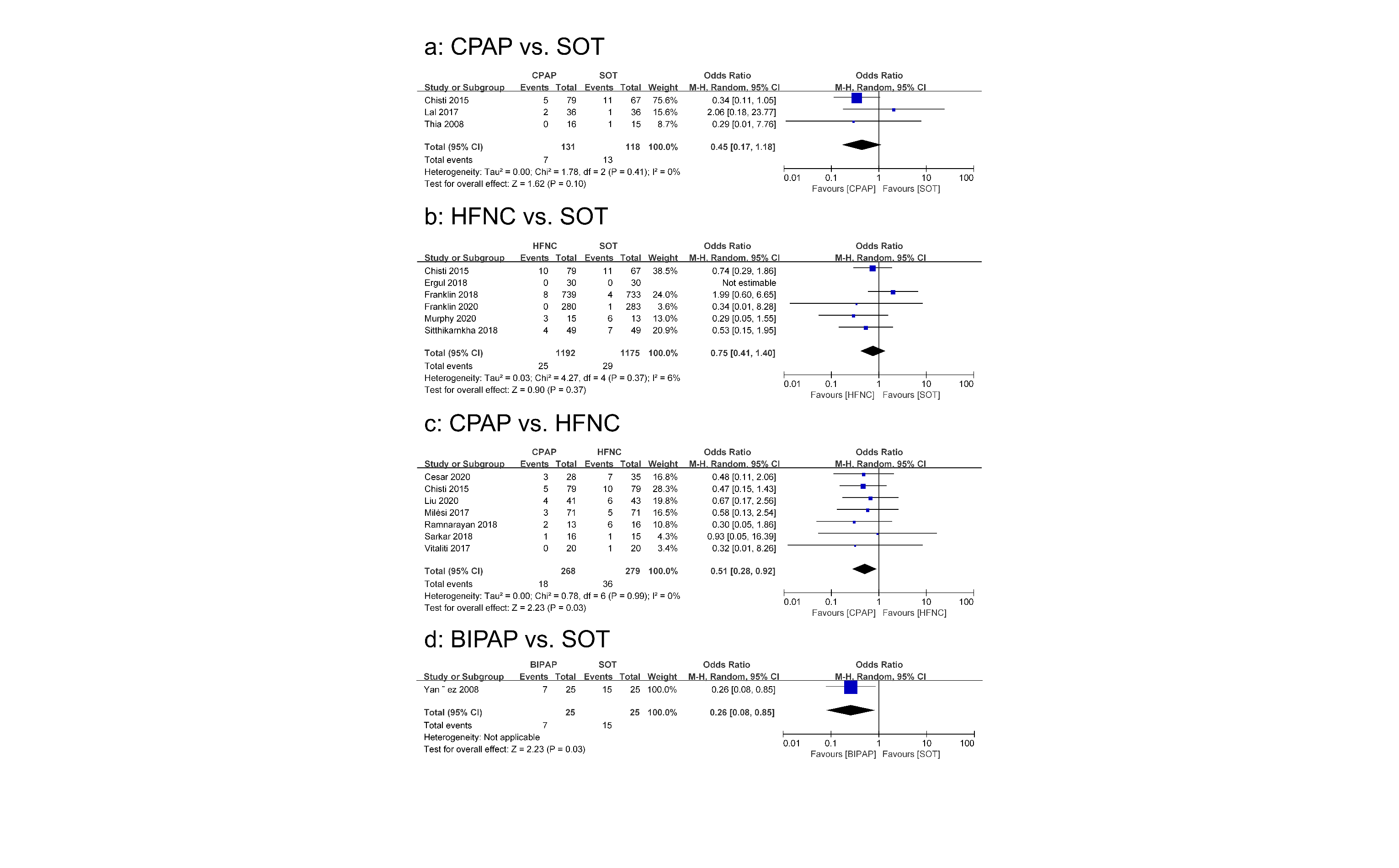


# Supplementary Figure 4. Forest plots for the pairwise comparison of in-hospital mortality. (a) CPAP vs. SOT. (b) HFNC vs. SOT. (c) CPAP vs. HFNC. CPAP: continuous positive airway pressure, HFNC: high-flow nasal cannula, SOT: standard oxygen therapy
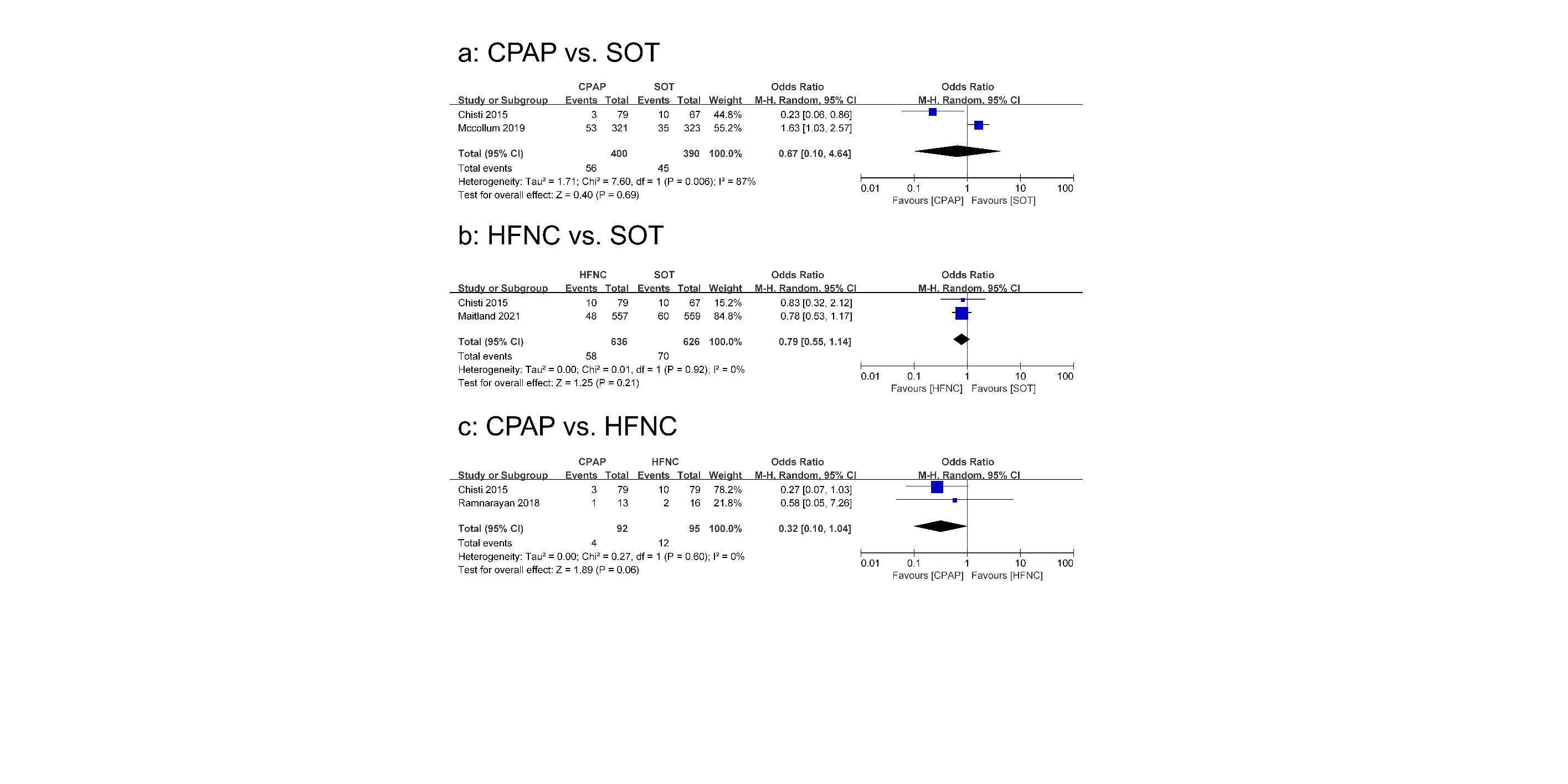


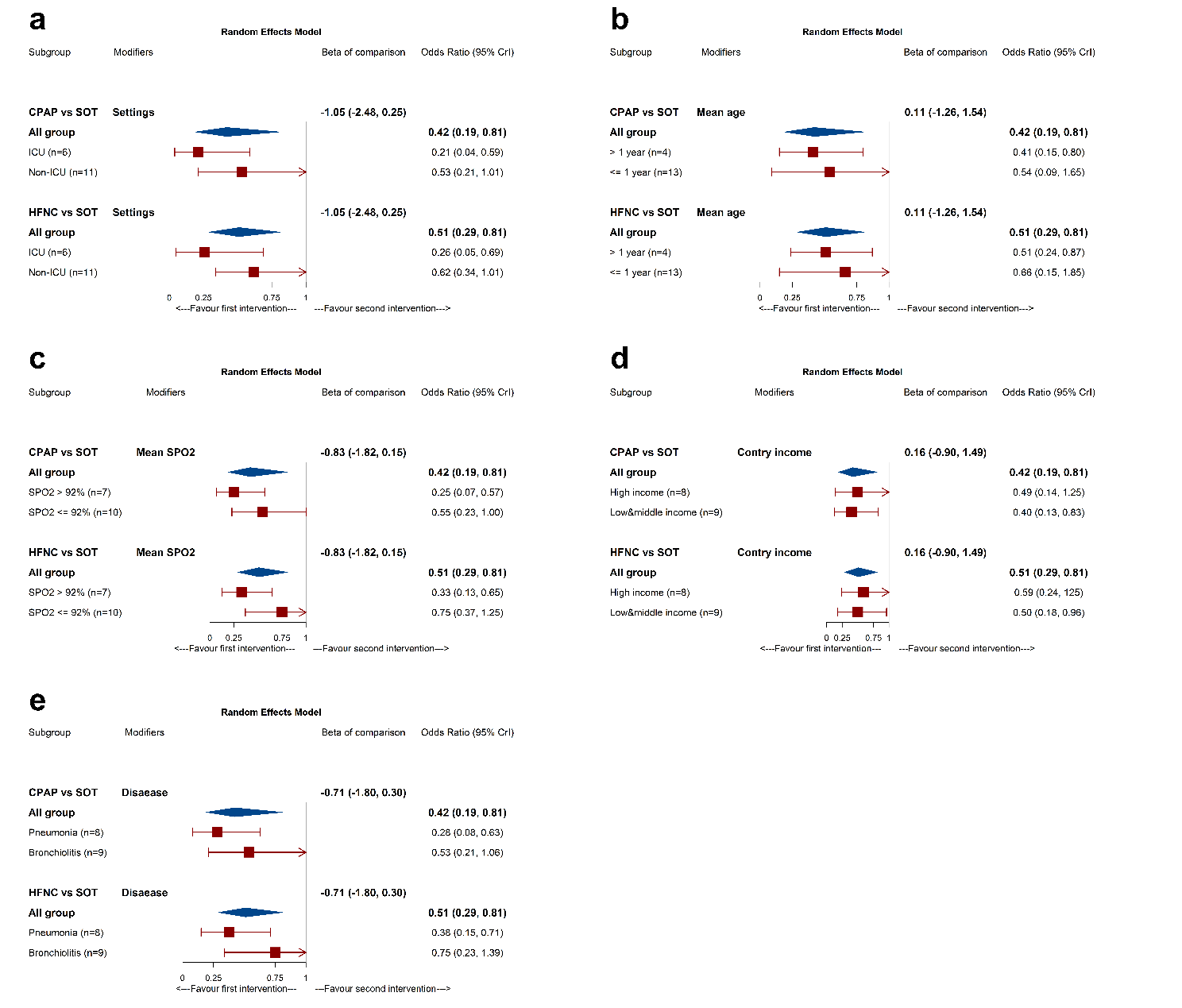


# Supplementary Figure 5. Subgroup and network meta-regression analyses for treatment failure compared with SOT.

# Subgroup and meta-regression based on: (a) study settings (b) mean age (c) mean SpO_2_ (d) contry income and (e) type of disease

* 95% Crls (Cridible intervals) of Beta-coefficient do not include 0 indicates significant subgroup effects.

ORs: odd ratios, 95% Crls (Cridible intervals), CPAP: continuous positive airway pressure, HFNC: high-flow nasal cannula, SOT: standard oxygen therapy


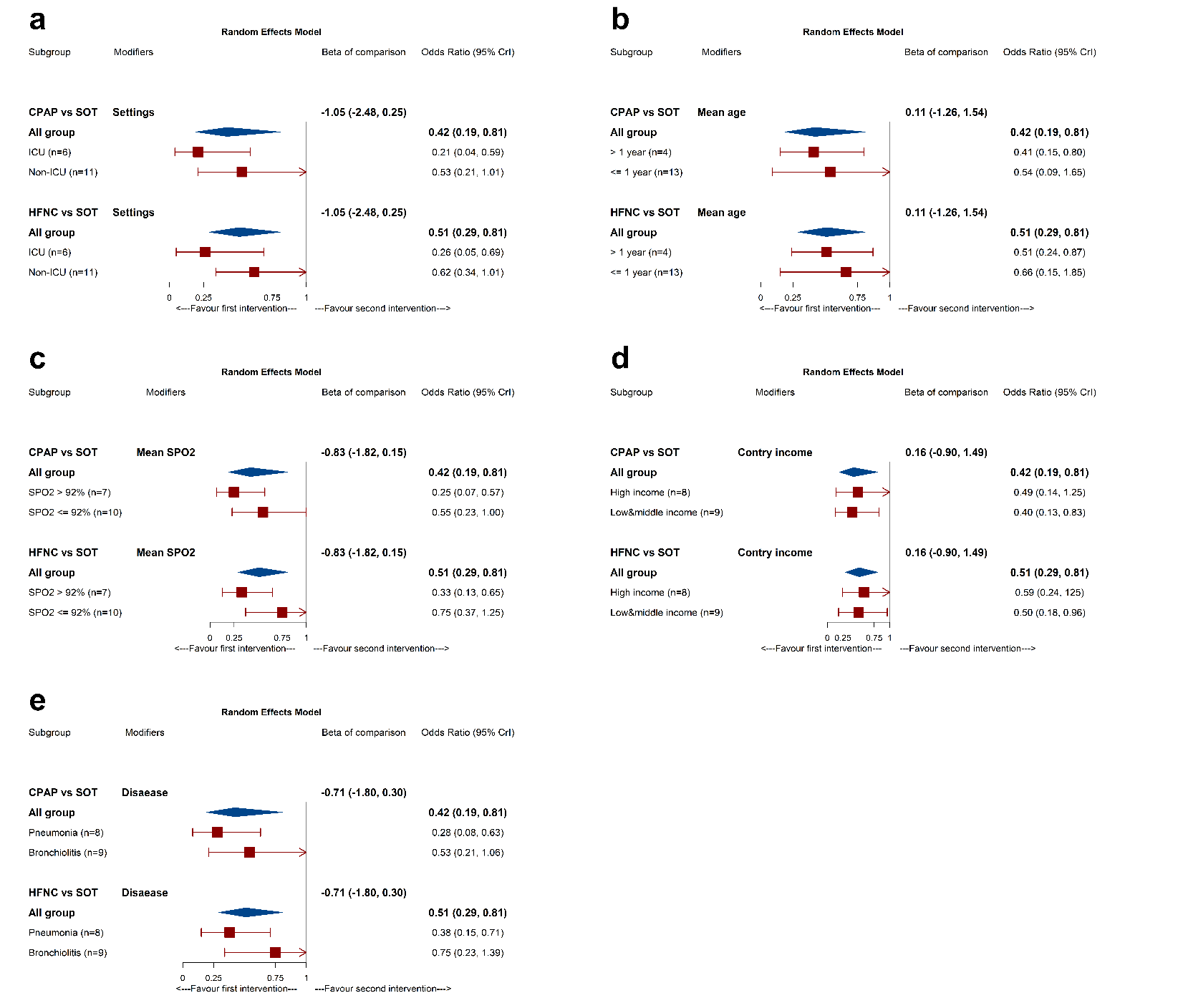


# Supplementary Figure 6. **Subgroup and network meta-regression analyses for intubation rate compared with SOT.**

Subgroup and meta-regression based on: (a) study settings (b) mean age (c) mean SpO_2_ (d) contry income and (e) type of disease

* 95% Crls (Cridible intervals) of Beta-coefficient do not include 0 indicates significant subgroup effects.

ORs: odd ratios, 95% Crls (Cridible intervals), BIPAP: bilevel positive airway pressure, CPAP: continuous positive airway pressure, HFNC: high-flow nasal cannula, SOT: standard oxygen therapy


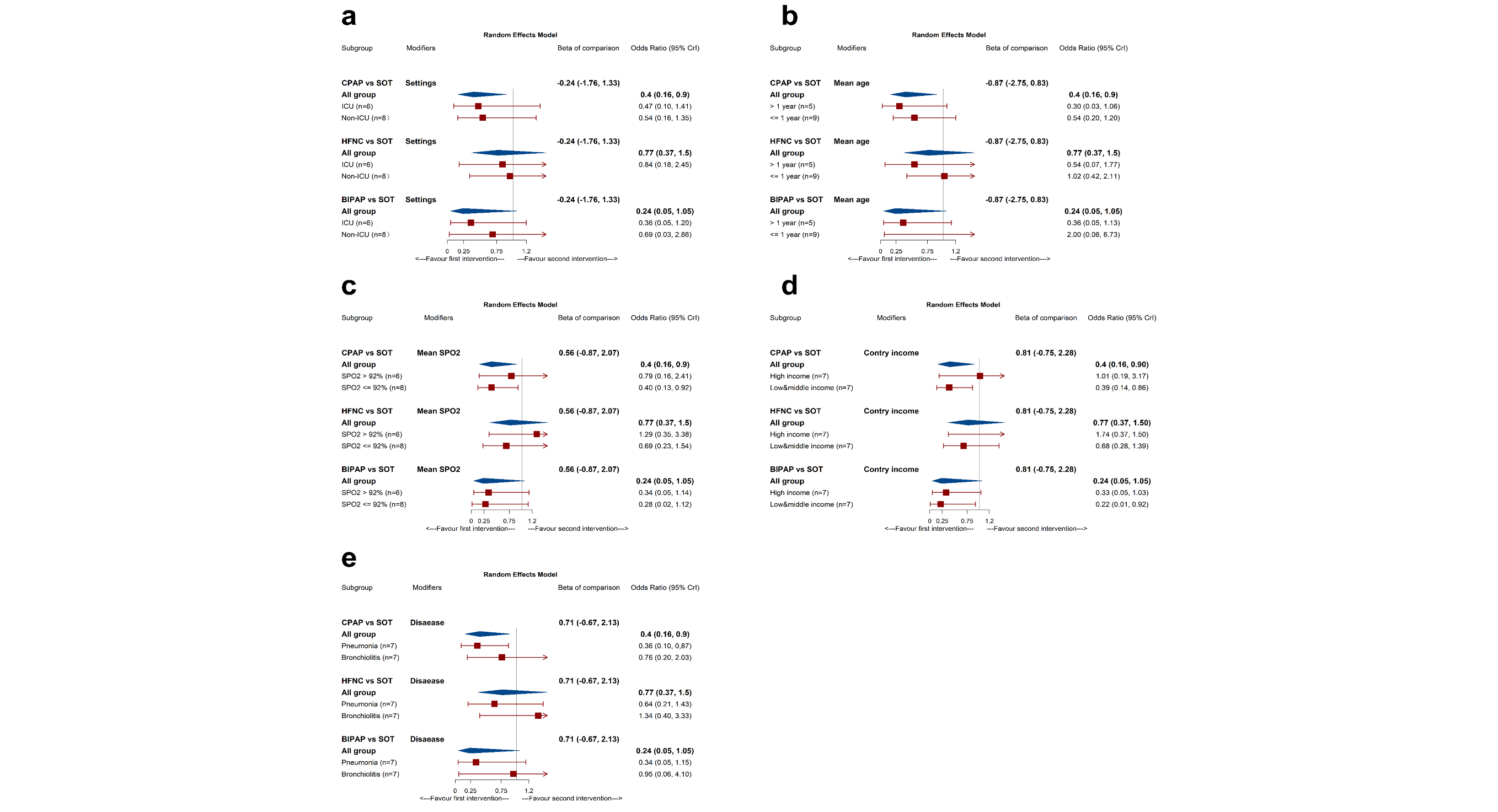


**Supplementary Figure 7.** Evaluation of the inconsistency heterogeneity for treatment failure by node-splitting method. (a) inconsistency assessments by node-splitting method, (b) heterogeneity assessments


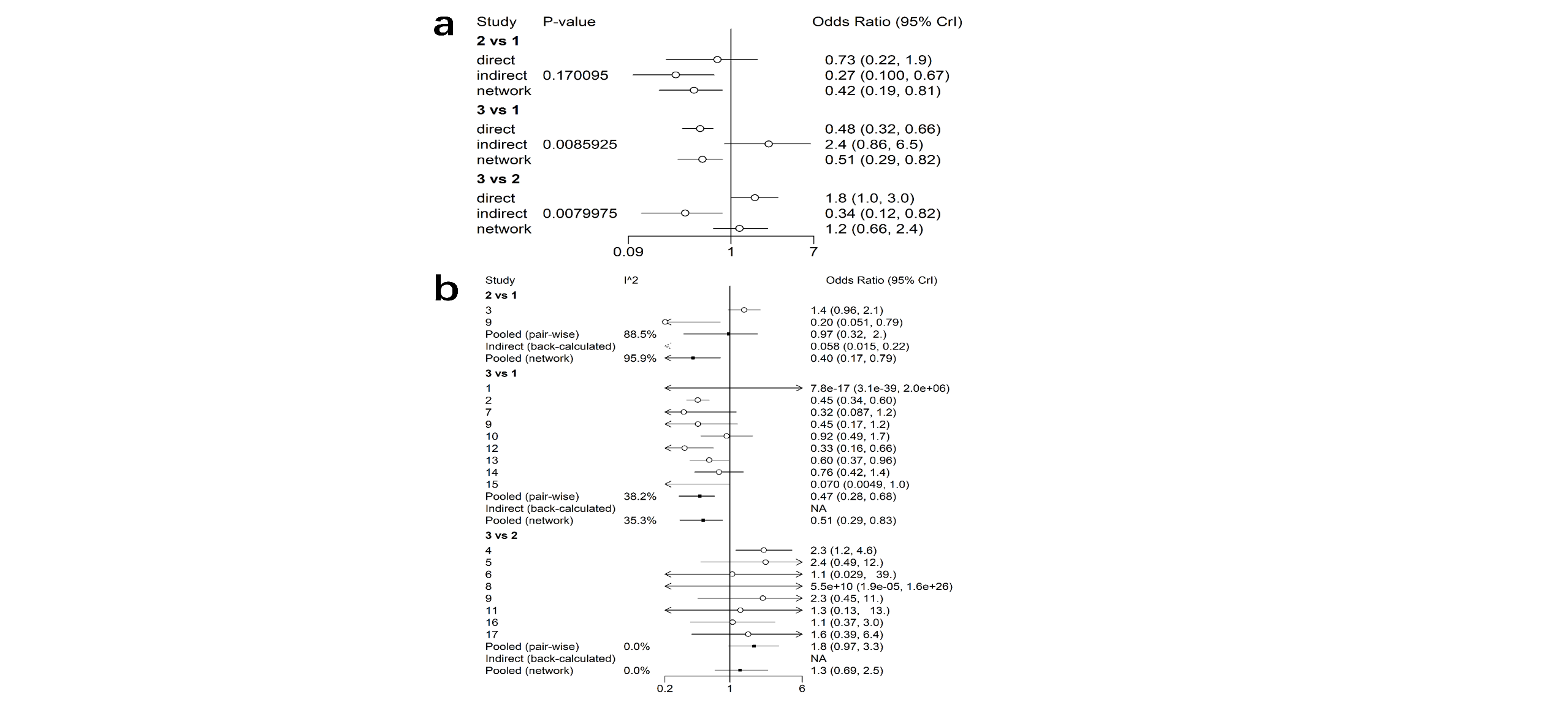


**Supplementary Figure 8.** Evaluation of the inconsistency heterogeneity for intubation rate by node-splitting method. (a) inconsistency assessments by node-splitting method, (b) heterogeneity assessments


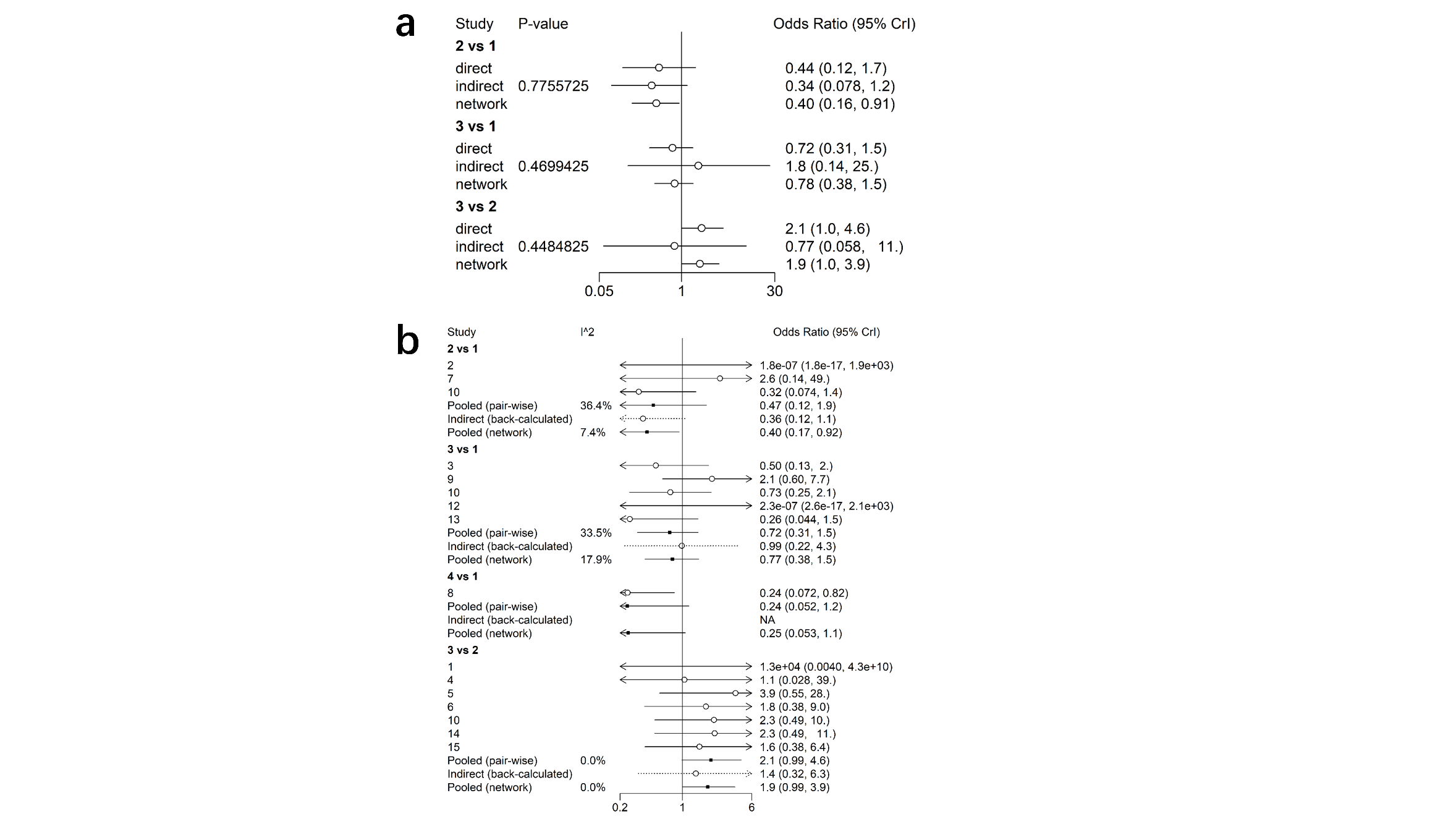


**Supplementary Figure 9.** Evaluation of the inconsistency heterogeneity for mortality by node-splitting method. (a) inconsistency assessments by node-splitting method, (b) heterogeneity assessments


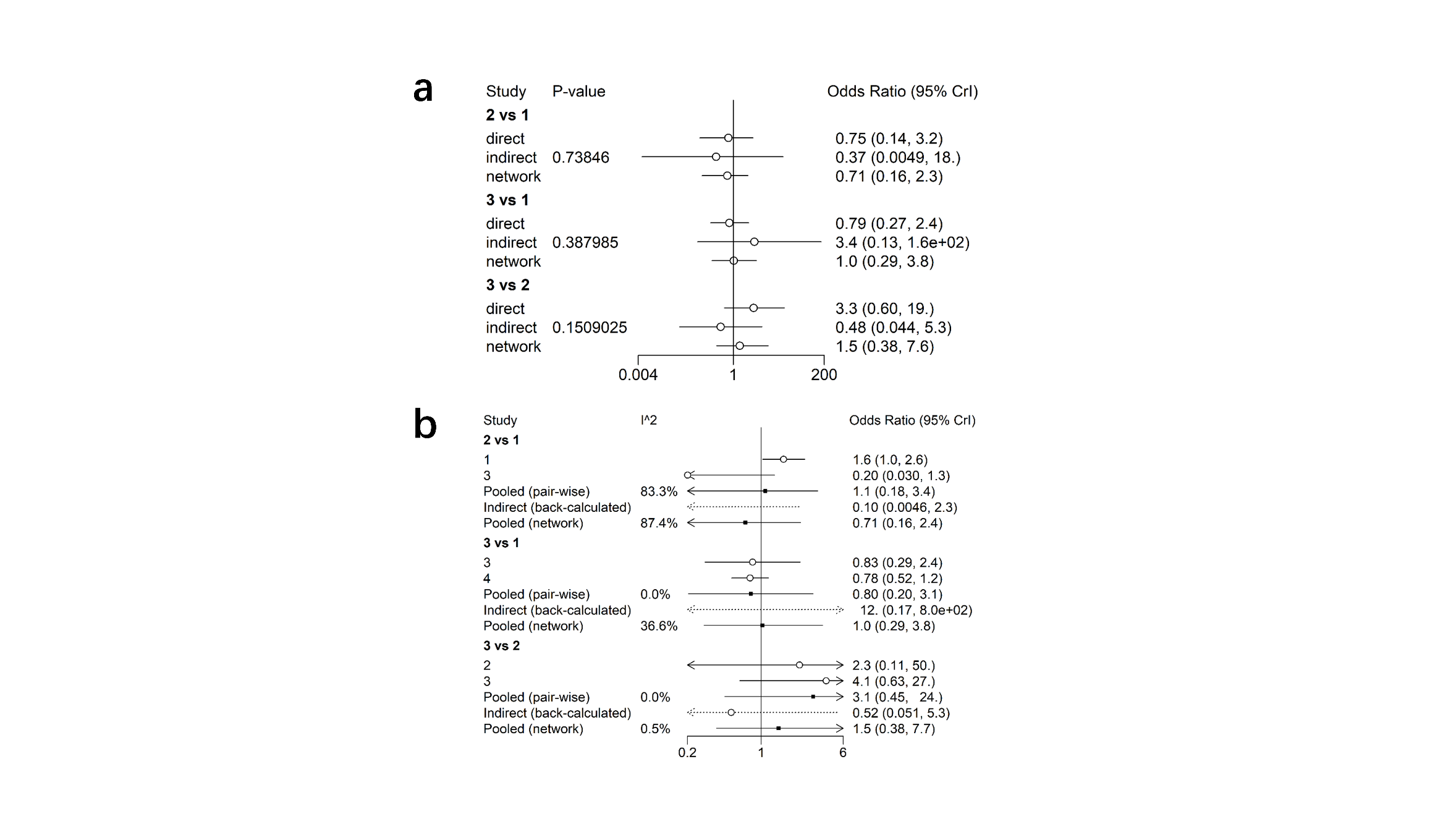


Supplementary Figure 10. Comparison-adjusted funnel plot. (a) treatment failure, (b) intubation rate. BIPAP: bilevel positive airway pressure, CPAP: continuous positive airway pressure, HFNC: high-flow nasal cannula, SOT: standard oxygen therapy


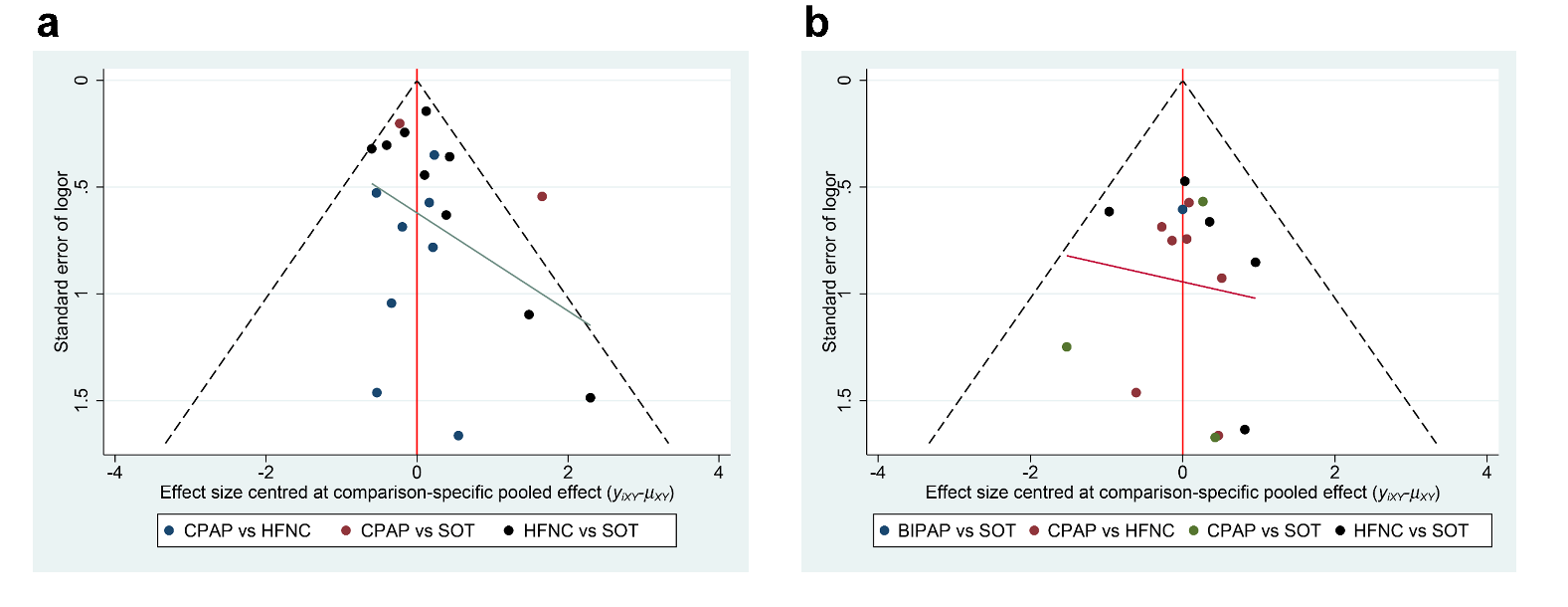

Supplement: Supplementary file 1 [file Data_Sheet_1.docx]
